# Willingness to Comply With Biosecurity in Livestock Facilities: Evidence From Experimental Simulations

**DOI:** 10.3389/fvets.2019.00156

**Published:** 2019-06-04

**Authors:** Scott C. Merrill, Susan Moegenburg, Christopher J. Koliba, Asim Zia, Luke Trinity, Eric Clark, Gabriela Bucini, Serge Wiltshire, Timothy Sellnow, Deanna Sellnow, Julia M. Smith

**Affiliations:** ^1^Department of Plant and Soil Science, University of Vermont, Burlington, VT, United States; ^2^Department of Community Development and Applied Economics, University of Vermont, Burlington, VT, United States; ^3^Department of Mathematics & Statistics, University of Vermont, Burlington, VT, United States; ^4^The Vermont Complex Systems Center, University of Vermont, Burlington, VT, United States; ^5^Department of Food Systems, University of Vermont, Burlington, VT, United States; ^6^Nicholson School of Communication and Media, University of Central Florida, Orlando, FL, United States; ^7^Department of Animal and Veterinary Sciences, University of Vermont, Burlington, VT, United States

**Keywords:** biosecurity, compliance, risk, uncertainty, graphical message, linguistic phrase, numeric message, psychological distance

## Abstract

Disease in U.S. animal livestock industries annually costs over a billion dollars. Adoption and compliance with biosecurity practices is necessary to successfully reduce the risk of disease introduction or spread. Yet, a variety of human behaviors, such as the urge to minimize time costs, may induce non-compliance with biosecurity practices. Utilizing a “serious gaming” approach, we examine how information about infection risk impacts compliance with biosecurity practices. We sought to understand how simulated environments affected compliance behavior with treatments that varied using three factors: (1) the risk of acquiring an infection, (2) the delivery method of the infection risk message (numerical, linguistic and graphical), and (3) the certainty of the infection risk information. Here we show that compliance is influenced by message delivery methodology, with numeric, linguistic, and graphical messages showing increasing efficacy, respectively. Moreover, increased situational uncertainty and increased risk were correlated with increases in compliance behavior. These results provide insight toward developing messages that are more effective and provide tools that will allow managers of livestock facilities and policy makers to nudge behavior toward more disease resilient systems via greater compliance with biosecurity practices.

## Introduction

Livestock diseases threaten animal welfare and livelihoods throughout production networks. Yet, due to the continued consolidation of livestock production (1, 2), increased movements of animals (3), and globalization of trade within livestock industries (4), disease prevalence is growing. For example, Porcine Epidemic Diarrhea virus (PEDv) was first detected in the U.S. in 2013 and within 12 months this disease had spread to 33 states. Nearly half of hog facilities have experienced a PEDv outbreak since its introduction (5) with net annual economic welfare reductions initially estimated at between $900 million to $1.8 billion (6). Many industry experts agree that implementing biosecurity practices is key to reducing the social and economic impacts of livestock diseases to industry stakeholder and consumers of hog products (7, 8), yet investment in biosecurity is low (9–11). Finding innovative and cost-effective ways to motivate, or nudge, farms to implement and comply with biosecurity practices will be instrumental in protecting livestock herds from endemic, exotic, and emerging diseases.

Biosecurity implementation at livestock facilities is both a tactical decision, with facility managers deciding which preventive biosecurity practices to adopt, as well as a series of ongoing operational decisions, with personnel deciding to comply or not comply with biosecurity practices (12). Regular compliance with biosecurity practices can significantly reduce disease. Unfortunately, many behaviors and signals in the work environment can negatively affect compliance. Behaviors such as habits, complacency, and the urge to minimize expenditures of time may induce non-compliance with biosecurity practices. For example, Beloeil et al. (13) found that consistently changing clothes before entering a hog facility reduced *Salmonella* seropositive animals from 87 to 13%. Yet studies have shown consistency in compliance with these practices is rarely the case (14). Racicot et al. (15), using hidden cameras, found 44 different biosecurity lapses made by workers and visitors at Quebec poultry farms over the course of 4 weeks, with the average number of biosecurity breaches being four per visit (15). The factors involved in this human behavioral dimension of biosecurity implementation are not well-known, but are thought to be essential to biosecurity practices and livestock welfare (16).

In this study we examine factors that may influence perception of disease risk and thus, compliance with biosecurity practices associated with production facilities. Variables to be treated in this study include: information regarding disease infection risk, amount of uncertainty associated with the information provided about the disease infection risk, and the types of message delivery methods used to communicate disease infection risk. These factors have been identified as influencing biosecurity in a variety of livestock systems (8, 17). Risk of disease is ubiquitous in livestock production facilities, but the level of risk at any given time is generally not known since it is challenging to quantify, and the sharing of information about disease prevalence and location is not standard procedure. Risk tolerance is generally high among U.S. farmers (18), but as the actual risk of disease infection is perceived to increase, farmers may be more apt to implement biosecurity or comply with biosecurity protocols (8, 19–21).

The degree of certainty provided in the infection risk message is also expected to affect biosecurity compliance. Ritter et al. (8) suggested that as certainty about disease information is enhanced (and uncertainty reduced), the benefits of practicing biosecurity are presented with greater certainty (19) or salience (22). This could lead to increased biosecurity implementation. Indeed, Merrill et al. (21) found, in an experimental simulation of disease in a swine production region, that a decrease in uncertainty (e.g., increase in certainty) about disease in a simulated swine production system was associated with increased tactical investments in biosecurity.

In addition to infection risk certainty, another important factor may be the perceived reliability of the information, which may vary depending on who is delivering it, consistency of messaging, and the mode of delivery (8, 16). Farmers in Ireland, for instance, received different information from veterinary practitioners vs. dairy advisors, and this perceived inconsistency or unreliability was stated as the primary reason for not implementing biosecurity, even while 83% of the farmers surveyed stated they would adopt practices if that would result in better herd health (23).

Another factor affecting compliance with biosecurity practices is the types of messages that workers at production facilities receive about disease, including the message delivery format. Evidence suggests that using impactful imagery should be more effective than using a number or phrase to convey messages (24). For example, since 1974 the U.S. government has used a rating system with five levels to inform people about the risk of wildland fires on public lands^1^ They convey this rating system using a threat gauge with an arrow pointing to a colored wedge of half of a wheel, with wedges labeled from “Low” to “Extreme” and colored green to red, respectively. This imagery effectively imparts the risk of a forest fire given the current environmental conditions, and evidence suggests that their use of a threat gauge is more likely to reduce dangerous fire behavior than simply using a phrase “Low” to “Extreme” or using some numerical equivalent. Thus, information delivery may be formatted to maximize reception and nudge workers toward greater compliance (22). Here, we examine the use of numbers (percentages), linguistic phrases (e.g., “Low Infection Risk”), and graphical imagery to pass information about the risk that the participant's behavior could result in their animals becoming infected with a disease. Because humans frequently use mental shortcuts, or heuristics, to calculate costs associated with risks, the way the message about infection risk is delivered impacts their decisions (25, 26). By design these shortcuts are quick and are largely based on experience, but they have the capacity to misinform because they perform poorly when experience is lacking, rely on affect or feelings, and do not rely on the heavy use of analytical reasoning (27).

Finally, economic factors and experience with disease likely play critical roles in biosecurity implementation decisions. Biosecurity adoption or compliance may be economically constrained in either direct costs, because biosecurity investments can be quite costly, or in indirect costs, such as opportunity costs, where time pressure has been cited as a major factor leading to lapses in biosecurity practices (28). Evidence suggests that recent experience with a disease outbreak will temporarily increase biosecurity practices (29), although increased biosecurity effort is not ubiquitous (30).

Understanding of decision-making processes has long been gained using economic experiments in controlled environments. Because these experiments are used to gather data, and frequently are couched with the idea of optimizing payouts, they broadly may be defined as serious games—games designed not for enjoyment but rather to gather data or for non-entertainment purposes such as education. While not always labeled as serious games, experimental economic games were pioneered in the work of Nobel Laureate Vernon L. Smith (31, 32) and multiple price list experiments (33, 34). More recently, experiments run on computers and as video games have become increasingly popular across disciplines, including adolescent risk behaviors (35), cognitive ability enhancement (36), and conflict resolution (37). The highly controlled environment within a computer game allows for testing of behavioral differences between, for example, genders (38) and among populations (39) or how individuals behave with increased informational awareness (21). Carefully constructed games provide insight into players' strategic, tactical, and mechanical decisions (40). Importantly, results from experimental computer games have been shown to mirror those from more traditional research instruments such as surveys [e.g., (35)] and, in some cases, games bring about better learning and retention than more traditional teaching methods [e.g., face-to-face, classroom settings or lectures; (41)]. Moreover, serious games offer the possibility of performance-based incentives. Performance-based incentives are used to make experiments more salient and increase effort in the decision making process, over more traditional information gathering techniques, such as surveys (42). As with many other serious game experiments (33, 35, 39), one of the primary sources of study participants was the university community.

Here we developed serious games to simulate a conflict where participants were confronted with a decision about avoidance or compliance with a common biosecurity practice: a “line of separation” at which workers shower and change clothing before entering or exiting areas with livestock. This line of separation is considered highly effective for reducing the risk of disease infection (7, 43). Use of a shower facility, however, carries opportunity costs associated with the time needed for its use, which can result in workers electing to bypass or only partially implement the practice.

We used various experimental scenarios to test participants' willingness to comply with the line of separation shower facility and incur associated opportunity costs in order to avoid potential direct costs associated with infected animals. Treatments varied the following factors: (1) the risk of acquiring an infection in their animals; (2) the certainty/uncertainty of the risk of infection by noting that the infection risk information provided as either a known value or a value that was uncertain; and (3) how the infection risk message was delivered, either with a linguistic phrase (Linguistic), a graphical threat gauge style image (Graphical) or a numerical (Numerical) value. We hypothesized (H1) that participants would become more compliant, and avoid risk as the infection risk increased (33, 44). Additionally, with increased uncertainty associated with the infection risk information, we hypothesized (H2) that we would see more compliance. Finally, we hypothesized (H3) that we would observe the highest compliance rates with infection risk information delivered using a graphical method, then using a linguistic phrase and finally the lowest compliance with infection risk messages delivered using a numeric value.

In addition to the three primary drivers noted above, we looked for secondary behavioral drivers. We posited that participants may immediately react to the economic consequences that result from their animals becoming infected by becoming more prone to comply with the shower facility biosecurity practice, but the tendency toward compliance may diminish with increasing time since the event (44–46). This effect is sometimes referred to as psychological distancing, hyperbolic discounting, or temporal discounting; here we refer to it as a psychological distance effect. We hypothesize (H4) that compliance would decrease with increased time since experiencing an infection.

## Methods

During early phases of this project, research team members met with biosecurity leaders to better understand how farm managers and farm workers make tactical and operational biosecurity decisions and discussed the biosecurity challenges faced by the industry. One outcome of these meetings was an increased awareness that compliance with biosecurity protocols was a serious problem. To study how decisions were made in the domain of biosecurity compliance, we developed two serious games that helped us capture the operational compliance dimensions of livestock biosecurity systems.

### Recruitment

We conducted two experiments using serious games to examine behavioral responses to variations in risk messaging. Experiment One was performed entirely at facilities on the University of Vermont campus. Experiment One participants were recruited using Craigslist, University listservs, direct emails, posters, and word of mouth. Approximately, 45% of recruits in Experiment Two were recruited as in Experiment One and through on-campus workshops, and ~55% were recruited through the online workplace Amazon Mechanical Turks (47). The research team recruited participants from the general public that were at least 18 years old. Because much of the participation was on the University of Vermont campus, many participants were graduate or undergraduate students (see limitations section Limitations). Recruits were told that they would be paid based on their performance during the experiment. Prior to beginning the experiments, participants were shown an informational slideshow that explained the purpose of the study. They were then shown a demonstration of the serious game, which was followed by a screen that gave them the choice to either proceed to game play, or exit and not participate. Institutional Review Board protocols were followed for an experiment using human participants (University of Vermont IRB # CHRBSS-15-319-IRB).

### Experimental Design and Development

**Figure 1:**
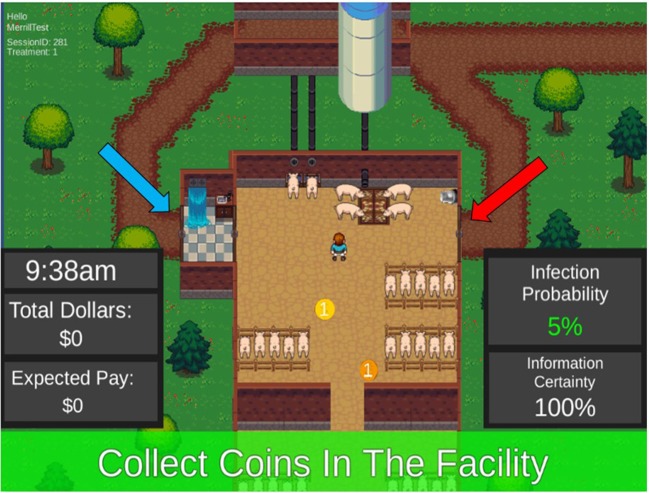
Screen view of a game round showing infection risk and uncertainty information, the worker and coins (internal tasks) inside the barn, the shower facility (blue arrow) and the emergency exit (red arrow), and the paths taken to attend to outside tasks. During each round, participants collect coins within the barn, then receive a cue to complete an outside task. The participant would then make a decision to use the shower facility or the emergency exit to complete the outside task based on the information provided. After completing the outside task (by touching the outside task space), the participant would return to the barn and collect more coins before the end of the working day.

The serious game platform used to run both experiments was developed using Unity software (Figure 1) (Unity Technologies, Version 5.3.5f1). Participants acted as workers in a simulated swine production facility and were confronted with experimental treatments that differed by the risk of infection if they broke protocol and exited through the emergency exit, and the information presented to them about the infection risk. Each round of the serious game represented one work day from 9 am to 5 pm. Both experiments included 24 rounds of play in addition to one practice round before the start of incentivized play.

The virtual farm worker was controlled by the participant using the arrow keys on a computer keyboard. Each round began with the virtual worker inside the barn, with tasks inside the barn represented as spinning coins; when the worker was moved to a coin the participant earned one experimental dollar. Spinning coins appeared at a rate of one coin every 2 s. One time during each round a high-value outside task would appear: either a feed jam in the silo, a break in the water pipe, or the arrival of a delivery truck. Attending to these outside tasks earned the participant experimental dollars, depending on how quickly they accomplished them. Each of these external tasks started out with a $30 value that decayed by $1 each second. To earn experimental dollars from these high-value tasks, the player needed to leave the barn, and this involved the primary decision in the game: whether or not to use the “shower in—shower out” biosecurity practice.

To use the shower biosecurity practice, the virtual farm worker would enter the shower facility, activating a 5 s counter that simulated showering and changing clothes. Then the participant could exit the facility to attend to the task. The procedure would repeat upon their return to the barn; shower again (which took another 5 s), and then re-enter the facility. The decision to not comply with the biosecurity practice involved leaving through an emergency exit, which had no delay and no opportunity cost, but risked infection. The opportunity cost associated with complying with the shower process was estimated at $7.50 because of the time lost getting to the outside task and the loss of internal task coins during the return to the facility.

In both experiments, if participants chose to use the emergency exit instead of the shower in—shower out facility, they would reduce potential opportunity costs but risk direct losses because their animals could become infected. Infections resulted from a random draw, with the actual risk of infection quantified using the expected risk of infection information presented to the participant. With an infection, the participant's animals would “die,” the round would immediately end, and they would lose $50 experimental dollars and any accrued earnings from that round. If their pigs did not get infected, they continued play until the end of the workday.

When the round ended, the number of experimental dollars earned was displayed on the participant's screen. After completing 24 experimental rounds, participants answered a few survey questions, and then were shown a completion code and the amount of real money they had earned. In Experiment One, the conversion rate between experimental and real dollars was $35 experimental dollars to $1 U.S. Participants then showed their screen to one of the researchers and were paid. In Experiment Two, the conversion rate was either $35 experimental dollars to $1 U.S. for all in-person mediated games or $350 experimental dollars to $1 U.S. plus a base pay of $3.00 for Amazon Mechanical Turk Participants.

### Treatments and Variables

**Figure 2:**
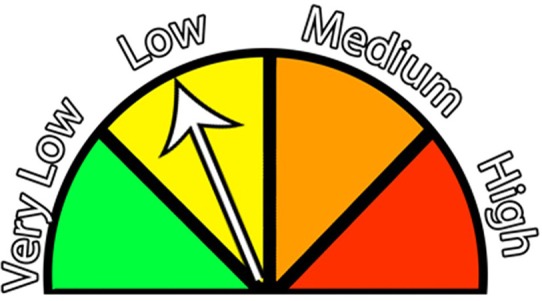
A threat gauge message format was used in Experiment Two's graphical message delivery treatments.

Early discussions with industry stakeholders highlighted that biosecurity was not simply a product of infrastructure and protocols, rather it was a product of infrastructure, protocols, and human behavior associated with willingness to use and comply with biosecurity protocols. Treatments were designed to test factors shown to influence willingness to comply, such as risk of loss, information uncertainty, and the media type used to deliver the message. In serious game play, participants received information estimating the infection risk and the uncertainty of that infection risk at the start of each round (Figure 2; Tables 1, 2). The infection risk information is used by the participant to either accept the risk by using the emergency exit, or accept the opportunity costs associated with the decision to use the shower exit. The infection risk message was delivered numerically (e.g., “5%” infection risk), linguistically (e.g., “low” infection risk), or graphically (i.e., using a threat gauge, Experiment Two only).

**Table 1:** Experiment one treatments.

**Treatment**	***N***
Infection Risk: 5% (Low)	944 (8 rounds*118 participants)
Infection Risk: 15% (Medium)	944 (8 rounds*118 participants)
Infection Risk: 25% (High)	944 (8 rounds*118 participants)
Uncertainty Treatment: Diagnosis Certainty: “100% Certain”	1,416 (12 rounds *118 participants)
Uncertainty Treatment: Diagnosis Uncertainty: “70% Certain”	1,416 (12 rounds *118 participants)
Message Delivery Method: Numeric: “1%,” “15%,” or “25%”	1,416 (12 rounds *118 participants)
Message Delivery Method: Linguistic: “Low,” “Medium,” or “High”	1,416 (12 rounds*118 participants)

**Table 2:** Experiment two treatments.

**Treatment**	***N***
Infection Risk: 1% (Very Low)	1,068 (6 rounds*178 participants)
Infection Risk: 5% (Low)	1,068 (6 rounds*178 participants)
Infection Risk: 15% (Medium)	1,068 (6 rounds*178 participants)
Infection Risk: 25% (High)	1,068 (6 rounds*178 participants)
Uncertainty Treatment: Contagion Certainty (Single best estimate)	2,124 (12 rounds *177 participants)
Uncertainty Treatment: Contagion Uncertainty (Best estimate plus a range of potential values)	2,124 (12 rounds*177 participants)
Message Delivery Method: Numeric: “1%,” “5%,” “15%,” or “25%”	2,124 (12 rounds*177 participants)
Message Delivery Method: Linguistic: “Very Low,” “Low,” “Medium,” or “High”	2,124 (12 rounds*177 participants)
Message Delivery Method: Graphical: (A threat gauge with arrows used to indicate risk)	2,124 (12 rounds*177 participants)

In Experiment One, participants received the Uncertainty Treatment in the form of a message describing the confidence in the diagnosis of the infection risk: A farm worker stating his level of certainty as either: “I have been working here for 30 years. I am 100% certain that there is an infection …” or “I just started working here. I am 70% certain that there is an infection …” While playing the round, the participant could see information about the Uncertainty Treatment by looking at a message displayed in the bottom right corner of the screen reporting “Information Certainty: 70%” or “Information Certainty: 100%” (Figure 1). Hereafter, this type of Uncertainty Treatment is referred to as Diagnosis Uncertainty.

In Experiment Two, participants received the Uncertainty Treatment using either a fixed level of infection risk or a variable level of infection risk. The message the participant received noted that the uncertainty was based on the understanding of the disease threat: “There is a well-known disease in your system with known contagion rates. There is a “low” probability of your animals getting sick if you leave through the emergency exit.” The following information was provided prior to starting each round “There is a poorly understood disease in your system. The best estimate is that there is a “low” probability (the probability could range from “very low to medium”) of your animals getting sick if you leave through the emergency exit.” As they were playing the round, participants could check the information about the Uncertainty Treatment through a display on the bottom right information box on the game play screen (Figure 1). Hereafter, this type of Uncertainty Treatment is referred to as Contagion Uncertainty.

Uncertainty Treatment messages were displayed either numerically (e.g., “1–15%”), linguistically (e.g., “low to high”) or graphically using a threat gauge (Figure 2, Experiment Two only).

In summary, after an initial examination of results from Experiment One, we noted that there was not a treatment that prompted a strong non-compliance signal (using the emergency exit a strong majority of the time), and thus, we decided to extend the treatment range in Experiment Two by adding an additional “Very Low” or 1% infection risk category. Additionally, discussions with stakeholders and others suggested the use of a graphical image to provide information was effective means of information delivery, and thus we added a graphical, threat gauge-style message delivery method. Thus, Experiment Two had four infection risk treatments that varied from very low (1%) to very high (25%) and three types of infection risk message delivery (Numeric, Linguistic, and Graphical). Both experiments had two Uncertainty Treatment categories related to the infection risk information (Uncertain and Certain).

Three additional variables were used in these experiments. First, because there were numerous rounds, we sought to control for within-experiment learning or behavioral trends (48, 49), and thus used a learning variable, referred to as Play Order, to account for within-experiment changes, such as tendencies to increase biosecurity as the experiment proceeded. Second, we used psychological distance to look for behavioral changes after participant's facilities became infected. Third, in Experiment Two, we used two different participant audience types. One audience group performed the experiment with a moderator present (from the Social Ecological Gaming and Simulation Laboratory, University of Vermont). The other group performed in the Amazon Mechanical Turks online environment (47). Moderated participants received a higher cash payout than those using the Amazon Mechanical Turk platform. These differences may have resulted in differences in salience between groups, and thus, an Audience covariate was included in the analyses.

### Analysis

A set of candidate models was developed to explain the decision response by the participants. All candidate models were mixed-effect logistic regressions models compiled using R statistical software (50, 51). Candidate models included treatment variables (Tables 1, 2), i.e., Infection Risk Treatments, Uncertainty Treatments, and Message Delivery Treatments, and interaction terms between treatments as well as the predictor variables: (1) psychological distance, (2) the learning variable: play order, and (3) in Experiment Two only Audience type. Participant was considered a random effect. Parameter estimation from mixed-effect binary logistic regressions are presented as logit coefficients that can be used to predict the probability (from 0 to 1) of a binary response, in this case, the probability that the participant would use the shower biosecurity facility given the combination of treatment information provided in the particular scenario. Logit coefficients can be exponentiated to generate odds ratios, which provide a measure of the odds that an individual will choose to use the shower biosecurity facility instead of the emergency exit. With odds ratios, a 1:1 ratio, presented as the value 1, indicates that there are even odds for the choice, and thus, if 1 is included in the odds ratio confidence interval, it may be equally possible that the participant will choose either the shower or the emergence exit and indicates that the variable does not have a significant signal.

One method to find the most parsimonious model when the number of possible combinations of explanatory variables is large is to create a set of plausible candidate models, and test to see how well each of them explains the data. We used an information-theoretic approach for candidate model selection (52, 53). Candidate model selection methodology and results are presented in Supplemental Material Appendix A.

## Results

### Experiment One

Data were collected from 118 participants in Experiment One. Fifty-four identified as female, 59 identified as male, and 5 choose not to identify with a gender. The mean age of the participants was approximately 25.4 years old. Payouts for this experiment averaged approximately $28 with a $19 minimum and a $37 maximum.

The AIC-selected best candidate model included Participant as a random effect and the fixed effects Infection Risk, Diagnosis Certainty, Message Delivery Method Psychological Distance and Play Order, as well as all interaction terms between Infection Risk, Diagnosis Certainty and Message Delivery Method (Supplemental Material Appendix A).

Logistic regression results presented in both experiments quantify the probability that a participant will comply with the shower biosecurity practice instead of using the emergency exit. Results presented in Table 3 are odds ratios. The top line represents the baseline odds ratio associated with the treatment combination of 5% Infection Risk, message delivered using a Linguistic phrase and with the Uncertainty Treatment set at 70% diagnosis certainty. The 3.337 odds ratio presented on the first line in Table 3 (intercept results) should be interpreted as participants are 3.337 times as likely to use the shower facility instead of using the emergency exit. The rest of Table 3 (i.e., excluding the top data line) are odds ratios compared with the baseline, intercept ratio. Odds ratio values in the table should be interpreted relative to the baseline treatment combination, i.e., the intercept value. For example, participants that received the infection risk information with a numeric message instead of the intercept value (Linguistic) had an odds ratio of 0.448, meaning they were 0.448 times more likely to use the shower door *than* the emergency door (or inversely 2.23 times more likely to use the emergency exit) than if they received information about the infection risk using a Linguistic phrase. If the odds ratio confidence interval includes 1 then it is unclear if or how the predictor variable will affect their decision to comply with the shower practice, and thus, variables that do not have 1 included in their confidence interval are significant variables. Odds ratios <1 indicate that it is relatively more likely that the participant will choose to exit through the emergency exit, whereas values above 1 indicate that they are more likely to comply with the suggested biosecurity practice by using the shower in—shower out door.

**Table 3:** Results of the selected best fit, mixed-effect logistic regression model (Model 8; see Table S1) for Experiment One.

**Parameter**	**Odds Ratio**	**Lower Bound**	**Upper bound**	***p-*values**
Intercept (Infection Risk @ 5%, Linguistic Message, Diagnosis Certainty @ 70)	3.337	1.811	6.149	** <0.001**
Diagnosis Certainty @ 100	0.206	0.132	0.321	** <0.001**
Psychological Distance	0.516	0.345	0.774	**0.001**
Numeric Message	0.448	0.291	0.690	** <0.001**
Infection Risk @ 15%	4.342	2.662	7.083	** <0.001**
Infection Risk @ 25%	5.223	3.168	8.612	** <0.001**
Order of Play	1.009	0.994	1.025	0.231
Diagnosis Certainty @ 100 by Numeric Message	1.451	0.775	2.716	0.245
Numeric Message by Infection Risk @ 15%	0.565	0.294	1.084	0.086
Numeric Message by Infection Risk @ 25%	0.868	0.446	1.692	0.678
Diagnosis Certainty @ 100 by Infection Risk @ 15%	8.152	3.937	16.883	** <0.001**
Diagnosis Certainty @ 100 by Infection Risk @ 25%	25.046	10.259	61.143	** <0.001**
Diagnosis Certainty @ 100 by Numeric Message by Infection Risk @ 15%	0.332	0.128	0.861	**0.023**
Diagnosis Certainty @ 100 by Numeric Message by Infection Risk @ 25%	0.451	0.147	1.389	0.166

*Depicted here are the odds ratios for the fixed effects describing relationships with the binary response variable: compliance with the biosecurity protocol. Bold values indicate significance at alpha = 0.05*.

**Figure 3:**
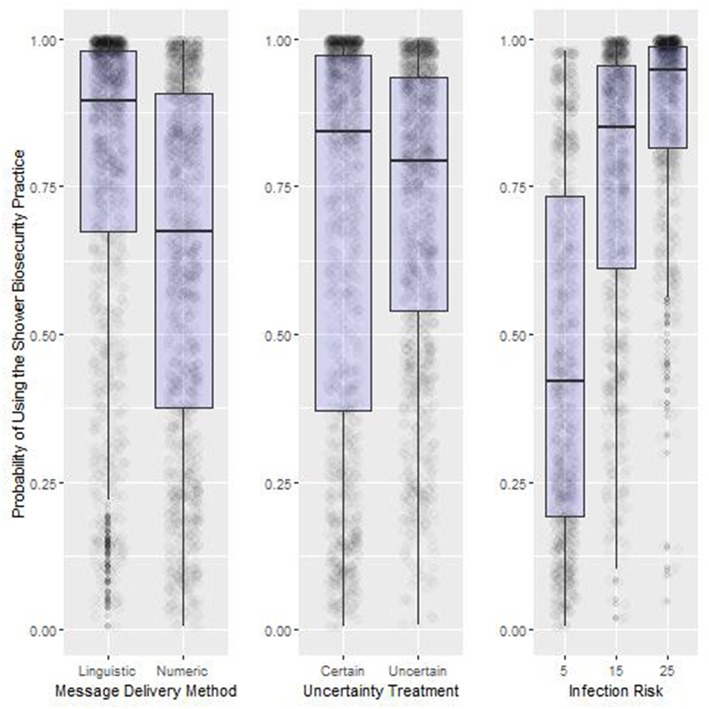
Summary results of the main treatment effects from Experiment One. Box-plot of the probability of using the shower biosecurity practice by the main effects, Message Delivery Method, Uncertainty Treatment, and Infection Risk, Lower, and upper box boundaries 25 and 75th percentiles, respectively, line inside box median, overlaid on model predicted data values.

**Figure 4:**
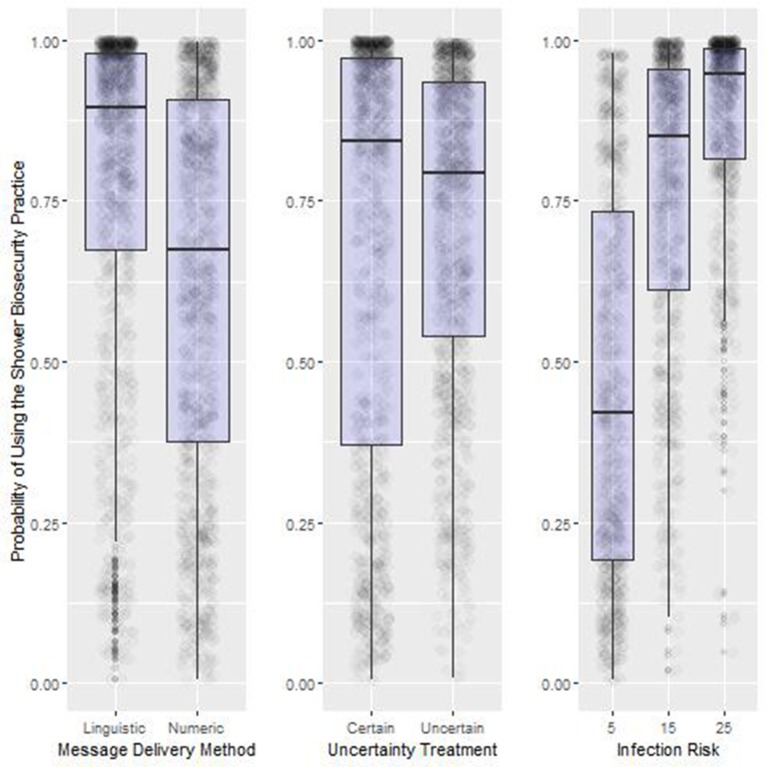
Summary results of the interaction effects between treatments from Experiment One. Box-plot of the probability of using the shower biosecurity practice by the interaction effects between Message Delivery Method, Uncertainty Treatment, and Infection Risk, Lower and upper box boundaries 25 and 75th percentiles, respectively, line inside box median, overlaid on model predicted data values.

We found significant main effects, two-way interactions and a three-way interaction between Message Delivery Method, Infection Risk and Diagnosis Certainty (Figures 3, 4, Table 3).

#### Main Effects (H1–H3)

Odds of compliance with the shower in-shower out biosecurity practice increased significantly as infection risk increased from 5% (46.4% compliance) to 15% (75.7% compliance) to 25% (93% compliance) (Figure 3 Right Panel). Shower use was much higher when the risk message was a linguistic phrase (77.7%) vs. numeric probability (62.3%) (Figure 3 Left Panel). Changing the Uncertainty Treatment from an message received from an advisor that was 70% certain of their report compared to 100% certain of their report resulted in a relatively small overall increase in the probability of observing participants using the shower biosecurity practice (Uncertainty: 71.6%. Certainty: 68.3%. Figure 3 Center Panel).

#### Interaction Effects (H1–H3)

Significant interactions with infection risk and message delivery type were observed (Tables 3, 4, Figure 4). The probability that participants would comply with the shower biosecurity depended upon the combination of treatments in the simulation with compliance values ranging from 31.2% with the treatment combination of 5% risk, message delivered numerically, and with 100% diagnosis certainty, to very high overall compliance when the message was delivered with certainty using a linguistic phrase (98.1%).

**Table 4:** Experiment One interaction affects.

**Infection risk (%)**	**Uncertainty treatment**	**Message delivery**	**Frequency of compliance (%)**
5	Certain	Linguistic	38.6
5	Certain	Numeric	31.2
5	Uncertain	Linguistic	65.8
5	Uncertain	Numeric	50.0
15	Certain	Linguistic	91.2
15	Certain	Numeric	61.4
15	Uncertain	Linguistic	84.9
15	Uncertain	Numeric	65.4
25	Certain	Linguistic	98.1
25	Certain	Numeric	89.5
25	Uncertain	Linguistic	87.5
25	Uncertain	Numeric	76.3

*Observed frequency of use of the shower in–shower out biosecurity practice*.

### Experiment Two

Of the 178 participants in Experiment Two, 76 were recruited as in Experiment One and through on-campus workshops, and 102 were recruited through Amazon Mechanical Turks. Ninety-nine identified as male, 76 identified as female, and three chose not to identify with a gender. The mean age of participants in Experiment Two was approximately 30.3 years old. Payouts for university community participants in Experiment Two averaged approximately $27 with a $16 minimum and a $35 maximum. Amazon Mechanical Turk participants received a base pay of $3.00 with additional performance bonuses ranging averaging $2.98, ranging from a minimum of $1.28 to a maximum of $3.84.

The AIC-selected best candidate model for Experiment Two included Participant as a random effect and the fixed effects Infection Risk, Diagnosis Certainty, Message Delivery Method, Psychological Distance and Audience, as well as the two-way interaction terms between Infection Risk and Message Delivery Method, and between Infection Risk and Contagion Certainty. Model selection results and details can be found in Supplemental Material Appendix A.

#### Main Effects (H1–H3)

**Figure 5:**
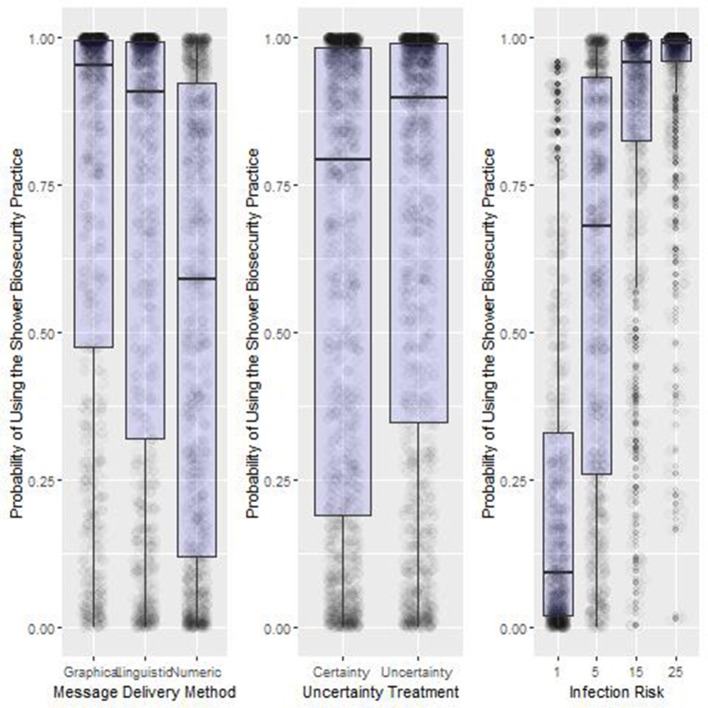
Summary results of the main treatment effects from Experiment Two. Box-plot of the probability of using the shower biosecurity practice by the main effects, Message Delivery Method, Uncertainty Treatment, and Infection Risk, Lower and upper box boundaries 25 and 75th percentiles, respectively, line inside box median, overlaid on model predicted data values.

As in Experiment One, odds ratios observed in Experiment Two confirmed the hypotheses about the main treatment effects (Table 5, Figure 5). Note that an odds ratio with a 95% confidence interval excluding the value 1 is considered significant with values <1 suggesting the odds of observing the shower behavior are less than the intercept, while treatments with odds ratio values above 1 are more likely to trigger compliance behavior. Inference from the selected best candidate model suggests that the use of the shower increased significantly as infection risk increased from 1% (23% compliance) to 5% (59% compliance) to 15% (85% compliance) and finally to 25% (93% compliance; Figure 5, Right Panel). Contagion Uncertainty, i.e., being unsure of the risk of acquiring an infectious disease with the use of the emergency exit, resulted in greater compliance with the shower biosecurity practice (Contagion Uncertainty: 69% compliance. Contagion Certainty: 62% compliance. Figure 5, Center Panel). Use of a Graphic (i.e., a threat gauge) to deliver the infection risk information message increased shower use over both the Linguistic and Numeric messages (Graphical delivery: 72% compliance; Linguistic delivery 69% compliance; Numeric delivery 60% compliance. Figure 5, Left Panel).

**Table 5:** Results of the selected best fit, mixed-effect logistic regression model (Model 2; see Table S2) for Experiment Two.

**Parameter**	**Odds ratio**	**Lower bound**	**Upper bound**	***P*-value**
Intercept (Contagion Certainty, Infection Probability @ 1%, and Graphical Message)	0.267	0.119	0.596	**0.001**
Contagion Uncertainty	1.173	0.795	1.732	0.421
Psychological Distance	0.574	0.339	0.970	**0.038**
Audience: SEGS Moderated	4.509	2.110	9.638	** <0.001**
Linguistic Message	0.365	0.208	0.639	** <0.001**
Numeric Message	0.270	0.156	0.468	** <0.001**
Infection Probability @ 5%	12.086	6.269	23.301	** <0.001**
Infection Probability @ 15%	352.507	144.614	859.261	** <0.001**
Infection Probability @ 25%	1571.303	438.113	5635.517	** <0.001**
Contagion Uncertainty by Infection Probability @ 5%	4.922	2.914	8.314	** <0.001**
Contagion Uncertainty by Infection Probability @ 15%	1.170	0.658	2.079	0.594
Contagion Uncertainty by Infection Probability @ 25%	1.010	0.500	2.040	0.977
Linguistic Message by Infection Probability @ 5%	1.040	0.493	2.193	0.918
Numeric message by Infection Probability @ 5%	0.399	0.196	0.816	**0.012**
Linguistic Message by Infection Probability @ 15%	0.934	0.355	2.463	0.891
Numeric message by Infection Probability @ 15%	0.196	0.079	0.489	** <0.001**
Linguistic Message by Infection Probability @ 25%	1.439	0.343	6.035	0.619
Numeric message by Infection Probability @ 25%	0.176	0.049	0.632	**0.008**

*Depicted here are the odds ratios for the fixed effects describing relationships with the binary response variable: compliance with the biosecurity protocol. Bold values indicate significance at alpha = 0.05*.

#### Interaction Affects (H1–H3)

**Figure 6:**
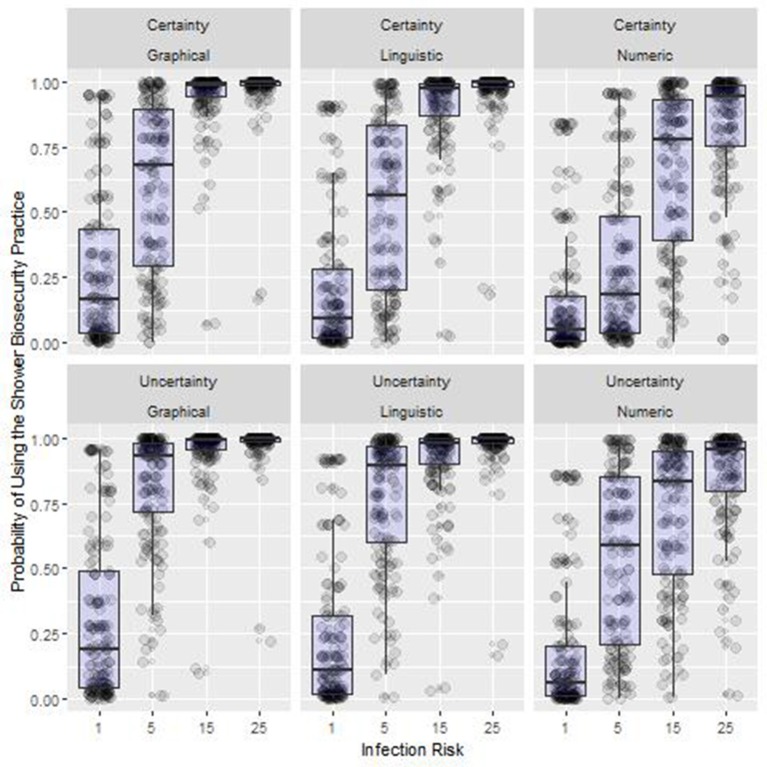
Summary results of the interaction effects between treatments from Experiment Two. Box-plot of the probability of using the shower biosecurity practice by the interaction effects between Message Delivery Method, Uncertainty Treatment, and Infection Risk, Lower and upper box boundaries 25 and 75th percentiles, respectively, line inside box median, overlaid on model predicted data values.

Substantial variation can be explained by the main effects, especially the infection risk treatment. Yet, the effects of these effects become more pronounced when we control for other variables in the system (Tables 5, 6, Figure 6). Compliance ranged from 16.7% with the treatment combination of 1% Infection Risk, message delivered numerically and with certainty, to the most frequent compliance at 98.1% when the Infection Risk was 25%, messages were delivered graphically and with uncertainty.

**Table 6:** Experiment Two interaction affects.

**Infection risk**	**Uncertainty treatment**	**Message delivery**	**Frequency of compliance (%)**
1	Certainty	Graphical	28.4
1	Certainty	Linguistic	21.9
1	Certainty	Numeric	16.7
1	Uncertainty	Graphical	30.4
1	Uncertainty	Linguistic	23.1
1	Uncertainty	Numeric	17.8
5	Certainty	Graphical	59.6
5	Certainty	Linguistic	53.0
5	Certainty	Numeric	30.0
5	Uncertainty	Graphical	82.0
5	Uncertainty	Linguistic	76.8
5	Uncertainty	Numeric	54.4
15	Certainty	Graphical	94.5
15	Certainty	Linguistic	90.0
15	Certainty	Numeric	66.1
15	Uncertainty	Graphical	95.7
15	Uncertainty	Linguistic	92.0
15	Uncertainty	Numeric	70.6
25	Certainty	Graphical	97.8
25	Certainty	Linguistic	97.3
25	Certainty	Numeric	83.9
25	Uncertainty	Graphical	98.1
25	Uncertainty	Linguistic	97.6
25	Uncertainty	Numeric	85.6

*Observed frequency of use of the shower in—shower out biosecurity practice*.

### Psychological Distance (H4)

In both experiments, evidence (odds ratios of 0.516 and 0.574 in Experiments One and Two, respectively), suggests that individuals are modifying their behavior based on the psychological distance by increasing the probability that they will comply with the biosecurity practice after becoming infected (Tables 3, 5). This was quantified by the number of rounds since they last experienced the economic consequences that results from their animal becoming infected.

### Learning Effect and Audience

In Experiment 1, the Play Order (learning) variable was selected for inclusion in the AIC-selected best candidate model, but it did not have support as a significant variable in the model. This indicates that it explained some of the variability in the data but did not have a consistent effect. Order of play was not selected as an important variable in Experiment Two.

A small difference in behavior was detected between the different participant types with Amazon Mechanical Turk participants taking more risks than participants at in experiment moderated by personnel from the Social Ecological Gaming and Simulation Laboratory (Amazon Mechanical Turks, 62% compliance. Moderated 69% compliance).

## Discussion

Common risks faced in agriculture often relate to the safety of the execution of tasks or the applications of products or technology. Farmers make decisions regularly on how to manage their farms, plants and animals and their risk perception influences their decisions (18). Risk messages are common and they aim at increasing awareness and/or understanding about the level of risk and motivating a behavioral change that reduces it.

The degree to which the risk information is received, its value and usefulness is affected by many factors including how it is framed and presented, and the recipient's situation/context at the delivery (54–58). In particular, the probabilistic dimension of risk is challenging to grasp for the general public. One line of research, inspired by risk communication theories, shows that the type of message chosen to convey probabilistic information (for example numeric, linguistic, graphical, or visual) has an effect on the degree to which perceived risk will change behavior (55, 56, 59). Overall, there is no one specific suggestion on which message format is best (55). The numeric format is precise and suggests scientific rigor but it may not connect with gut-level reactions in people who do not have familiarity with methematical concepts (low numeracy). The verbal format allows more fluid communication but it might lack precision. Graphical and visual formats have become common in conjunction with numeric or verbal risk messages because they can encapsulate data, patterns, and matematical relationships. The immediacy of graphical formats is very appealing but it needs the right skills and/or information for interpretation. Recomendations on how to improve messages of risk probability are suggested in the paper reviews by Lipkus (55) and Visschers et al. (56). Most reviews published on risk-probability communication are in the medical field. The need for guidance and research on effectively using risk messages in any form (visual, graphic, linguistic or numerical) is growing. There are still important questions about how to compose messages for best efficacy in the farming context. With our experiment, we aimed at understanding the effect of message type at the operational level of biosecurity in the hog farming system. In a recent publication, Merrill et al. (21) showed that the type of information provided to inform about disease risk in a hog production system affects the direction of behavioral change. Specifically, information on disease presence lead to increased investment in biosecurity. On the other hand, information about neighbors' biosecurity level triggered a free rider effect in a significant fraction of individuals. Our current study is therefore timely and essential to test and tailor risk messages in the specific context of biosecurity compliance in livestock production systems.

We examined effects of different aspects of infection risk information and delivery on compliance with biosecurity by testing for effects of disease infection risk, information uncertainty, and message delivery methods. As hypothesized, compliance with the biosecurity practice of using the shower facility increased significantly with increasing disease infection risk. With increased diagnosis and contagion uncertainty, participants increased the frequency of their safe choices (compliance with biosecurity). Graphical information in the form of a threat gauge increased compliance more than information delivered linguistically, with the least use of the shower facility associated with infection risk information delivered numerically. We also found evidence of psychological distancing—the more time that passed since participants experienced the consequences of their animals getting a disease, the less compliant they became.

### Limitations

Social, psychological and behavioral economics studies frequently use participants from a portion of society, such as a student population, and attempt to extrapolate to a larger or more diverse group. This could lead to bias if the participants behave differently than the population of interest. Participants in the present study were not recruited based on experience working in swine production facilities and thus, participant behavior may not accurately reflect worker behavior. For example, U.S. farmers are thought to be relatively more risk seeking than the general public (18). However, Zia et al. (60), using a similar serious game methodology to study risk and information in the swine industry, did not find a significant difference between swine industry stakeholders and behavior of those not known to have any experience in the industry. Their conclusions should be taken with some caution because their sample population of ~100 participants may not have been large enough to detect a difference in the behavior between populations. People are complex and make decisions based on a number of rational and irrational factors. Because of this complexity, true differences in the decision process between population groups may be hard to tease out. Moreover, experience will alter one's heuristics but may not do so consistently. Therefore, workers in the swine production industry may behave differently than participants selected for our study, yet because workers are complex and each have their own set of objectives, any bias that exists may not be consistent. In addition, we attempted to reduce the potential for participant subset bias by using multiple, distinct participant group types (i.e., Amazon Mechanical Turk participants and the community recruited to participate on the University of Vermont campus). Using multiple groups allowed us to control for statistical differences among participant groups and should reduce bias that may be associated with a particular subset e.g., bias that could be generated if participants were from only a student population. Although, data from an all-worker participant population would be ideal, it is logistically impractical and good evidence exists (60) that behavioral differences between participant populations may be minimal.

### Infection Risk (H1)

The experiments were designed with PEDv in mind. The number of hog facilities in the U.S. was estimated at 69,100 in 2011^2^. At the height of the PEDv outbreak in 2014, the number of infected facilities reached over 1,200 (61), putting the probability of infection at approximately 1.75%. This value is close to the “very low” treatment in the present study, but when considered as a series of choices over the course of a week, reflects that the likelihood of an infection break per behavioral choice is exceptionally low. However, in aggregate, many choices can impact biosecurity and aggregate decisions may approximate the 1% treatment in Experiment Two. Moreover, for any given region during an outbreak, the probability may approach much higher numbers, which we reflect using our higher treatment levels of 15 to 25%.

Results of this study may extend for use in communicating to farm workers, nudging behavior toward greater biosecurity compliance, and thus, reducing their facility's risk (8). By design we varied the risk of infection if the participants chose to exit through the emergency exit. In the high risk treatments, we anticipated that most of the individuals would avoid risk by complying with the suggested biosecurity practice and conversely, we hypothesized that most would choose not to comply in the low risk scenarios. As hypothesized, we found significant jumps in compliance as the infection risk increased.

In Experiment One, less than half complied with the low (5%) Infection Risk treatments, around complied with the medium Infection Risk treatments and compliance approached 90% with the high Infection Risk Treatments. After running Experiment One, we recognized that we should include an additional very low risk category to prompt even more non-compliance behavior. Similar patterns were observed in Experiment Two with less frequent compliance (around a ¼) in the very low, 1% Infection Risk treatments, with an increase in the choice to use the biosecurity practice (over half complied) as the risk of infection increased to low, 5% Infection Risk treatments, about 85% compliance with the medium, 15% Infection Risk Treatments, and compliance over 90% with the high, 25% Infection Risk treatment. The wide range of compliance observed by treatment confirmed that our simulation was able to elicit a substantial range of behaviors, and thus observe how the treatments combined to observe emergent patterns of behavior. These findings are supported by the risk aversion literature (33, 34, 44).

### Infection Risk Uncertainty (H2)

Supporting previous research, we found evidence for an uncertainty aversion effect in both experiments (62). Participants were less apt to use the emergency exit to increase their payouts if they believed that the information about the risk associated with the behavior was uncertain. However, this effect was not exceptionally pronounced as a main effect because of the variation associated with infection risk and message delivery format. However, when we examine uncertainty when controlling for other variables, the effect is dramatic. In Experiment One, when infection risk was set at 5%, and message was delivered with certainty as the Linguistic phrase “Low,” participants chose to use the shower biosecurity practice 38.6% of the time, and 65.8% when the infection risk message was delivered with uncertainty, which marks over a 70% increase in compliance associated with uncertainty aversion. Similarly, in Experiment Two, with the Infection Risk at 5%, and numerical message delivery, 30.0% complied with biosecurity when infection risk information was certain, whereas 54.4% complied when there was uncertainty about infection risk. So in these two scenarios, by simply changing the level of certainty in the infection risk information, we altered compliance from ~1/3 probability of using the shower biosecurity to on average around 3/5 probability of compliance. These shifts suggest exceptional uncertainty aversion constrained by situational risk (62, 63). An exception to the uncertainty aversion effect appeared in Experiment One, when significant infection risk prompted a strong majority of participants to use the shower facility. In this case, adding uncertainty slightly reduced the likelihood of using the shower facility. This slight decrease may stem from the wording that could have been interpreted to mean “I believe the infection risk is high, but there is a chance it could be medium or low.” With this interpretation, uncertainty in this situation would produce a bias because the direction of uncertainty could only reduce the probability of infection.

### Message Delivery Format (H3)

Compliance with biosecurity protocols was observed more frequently when the infection risk was conveyed using a graphical representation (the threat gauge, Figure 2), followed by messages delivered using a linguistic phrase, with the least compliance when a numerical representation of the infection risk (Tables 3, 5). The effect of message delivery format becomes more pronounced when the other two treatment variables are controlled. In Experiment One, we observed the most extreme changes in behavior with the Infection Risk at 15%, and with messages delivered with certainty, 91.2% of participants used the shower biosecurity practice when the message was delivered using the linguistic phrase “Medium Risk” contrasted with 61.4% when the risk message was delivered numerically as “15%.” In Experiment Two, we observed the most dramatic behavioral shift associated with message delivery type with the treatment combination of Infection Risk at 5%, and the message delivered with certainty. With this treatment combination, there was a 59.6% probability that participants would comply when the message was delivered graphically, and a 30.0% compliance rate when the message was delivered numerically. These results corresponds well with previous research that suggests using graphical representations of information for efficient information transfer (42). Moreover, research has noted that people are notoriously poor at calculating cost loss functions using numerical probabilities (24, 44). Because we requested quick decisions for each scenario, we suggest that participants were using mental shortcuts to calculate the if the risk was worth the potential benefit, and in the high risk scenario the numeric risk was 25%. Compared to 100%, this value is relatively low, and experientially, many may have quickly considered the likelihood of an infection to be low without analytically assessing the relative benefit and relative costs of their decision (25, 27). In this case, there was a 75% chance of earning approximately an extra $9 experimental dollars and a 25% chance of losing around $80 experimental dollars. If participants had fully assessed those terms, it is unlikely many would have chosen to use the emergency exit. Moreover, Slovic et al. (25) suggest that the feeling, or affect, behind a decision can sometimes discount probabilities so that the decisions are made without fully analyzing the values but rather simply by assessing whether the decision “feels” risky. Thus, participants may have observed the numeric “15%” or “25%” and intuitively felt that the risk was low, and thus, made a gut decision to exit through the emergency exit. The graphical image, using a threat gauge, may have triggered an additional affect, associated with risk and prompted additional constrain over the simple linguistic phrase. These results reinforce the impact of the method of message delivery, because many of our quick decisions rely on mental shortcuts, based on previous experience (26, 27).

### Psychological Distance (H4)

Historic exposure to an infection ties in with the concept that humans use experientially-informed mental shortcuts to help make decisions. The relationship with passage of time and the influence of experience is captured in the concept of psychological distance. In both experiments participants that had just had animals infected because of their choice to use the emergency exit were approximately twice as likely to use the shower facility compared to those that were nearing the end of the experiment and had not experienced an infection. Thus, participants behaved with increasingly risky behavior as time passed since they had experienced an infection. This evidence for psychological distancing (45, 46, 64) has profound education implications for the industry because it reinforces the need for temporally frequent reminders or messages, especially those messages that support internalization of the material, explain the problem and provide appropriate actions (65), as well as assist in reduction of the discounting through reinforcement-learning (66).

### Selecting Appropriate Combinations for Effective Messaging

As noted above, the combination of treatments could change behavior from infrequent compliance to nearly ubiquitous compliance. Even without altering the actual Infection Risk, the rate of compliance with the shower in—shower out biosecurity practice could be dramatically shifted with messaging. For example, in Experiment One, with the treatment combination of 5% risk, numerical message delivery, and with 100% diagnosis certainty, we observed that just under a third (31.2%) of the participants complied by using the shower biosecurity practice. Yet almost two-thirds (65.8%) of participants complied when the message was delivered with uncertainty about the diagnosis and with the message delivered using a linguistic phrase. Within the subset of high infection risk treatments from Experiment One, when the message was delivered numerically with uncertainty, compliance was observed in ~76% of trials, whereas if the infection risk message was delivered using a linguistic phrase with certainty, we observed approximately 98% compliance with the shower biosecurity practice. This evidence suggests that we can influence behavior through explicit message design and delivery with the potential to radically change behavior, with an intended goal of changing the default behavior and the culture within the system (67).

Compliance with existing biosecurity practices is one of the critical issues that confronts managers in the swine industry (8). In August 2018, the authors were requested to run a workshop for industry professionals to help assist in designing messages to improve biosecurity compliance in their production system. In light of this workshop, we recognize that compliance is something that managers struggle with on a daily basis.

“I would be happy if I could get my guys to use soap.”–An industry professional at a workshop titled *Improving Workplace Compliance Through Message Development*. Minnesota, August 2018.

Human behavior in the animal livestock industry remains a challenge because of the serious ramifications of a disease outbreak and the ongoing fight against complacency (8, 15). Here we confirm that compliance with existing biosecurity measures may be influenced by the way that we provide information to those working in the facilities. Graphical messages that make note of the inherent uncertainties rather than numeric “best estimates” of the risk of contagion should provide the most reliable compliance with existing biosecurity rules. If graphical message delivery is not an option, our research provides evidence that the use of linguistic phrases should be encouraged over the use of numerical delivery. Costs for biosecurity failures can be exceptionally high. In addition to the direct costs associated with animal mortality and stunted growth, owner operators and their workers can experience the loss of livelihoods and even experience an array of mental health issues. Awareness that psychological discounting temporally (and likely spatially) will distract workers reinforces the idea that biosecurity communication and trainings need to be frequently reinforced.

## Conclusion

As we have noted, the types of factors that influence the abilities of farm workers to comply with biosecurity protocols like the shower-in-shower-out facilities simulated in these experiments are complex. Drawing on a number of factors that industry leaders have highlighted for the researchers, three possible influences were tested. The first is when the infection risk is communicated, we see that the higher the risk, the stronger the compliance.

The second influence is the level of certainty that the worker has in the risk that their behavior may lead to an infection. In Experiment One variations in this certainty as a matter of both trust in the experiences of an experienced farmer (who is 100% certain of his forecast) and the less trustworthy experiences of a new, less experienced farmer (who is only 70% certain of his forecast). Operationally, we can interpret this as the relative importance of the medium through which a risk message is conveyed. Our results demonstrate that the assuredness of information about the risk associated with behaviors may reduce the rates of compliance with biosecurity protocols. In Experiment Two, uncertainty was associated with epidemiological factors. Regardless of the type of uncertainty, increased uncertainty led to increased compliance. This result may seem counter-intuitive. However, the selection of infection risks examined were all relatively low from a pure probability perspective—at the maximum, infection risk was 25%. In general, people tend to discount the probability that something bad will happen if its likelihood is low (44). When that probability is more certain, this discount effect is more likely, because when a probability is less certain, the potential for even higher rates of probability are there, leading to increased risk aversion (62, 63, 68).

Thirdly, these findings confirm that the method by which risk is conveyed matters. Graphical displays are more effective than linguistic, followed by numerical displays.

If findings are extended to the farm level, the operational and policy implications from this study are summarized as follows. If routine compliance with biosecurity protocols is desirable, then one can expect significant variance in responses as risk threat is communicated to farm workers. These findings suggest that although risk communication is still likely very important, the active and ongoing reporting of risk threat, regardless of the medium (the assuredness of the messenger) and means of communication are influential and should be considered with the audience preferences in mind.

The findings of this project suggest that messages delivered using graphical means to convey disease infection risk, include infection risk uncertainty, and are delivered with relatively high frequency to reduce the psychological distancing effect, have the potential to dramatically improve biosecurity compliance on livestock facilities. While we acknowledge that idiosyncrasies in human nature will not disappear, we believe that even small improvements in compliance can have a profound impact in the reduction of disease, improving the welfare of animals and the livelihoods of workers across this industry.

## Ethics Statement

This study was carried out in accordance with the recommendations of the Collaborative Institutional Training Initiative via the Committee on Human Research Behavioral and Social Sciences at the Research Protections Office at the University of Vermont. The protocol was approved by the Committee on Human Research Behavioral and Social Sciences.

## Author Contributions

SCM, SM, CK, AZ, SW, LT, JS, EC, TS, and DS assisted with design and conceptualization of the experiment and underlying serious game. LT, EC, and SCM helped with data curation. SCM, SM, and GB worked on data analysis. Project funding was generated with the help of JS, SCM, CK, AZ, and TS. Experiments were conducted by SCM, SM, LT, and EC. Software development was primarily led by LT and EC. Initial manuscript drafts were created by SCM and SM. Subsequent manuscript editing and retooling was completed by all authors, SCM, SM, CK AZ, LT, EC, GB, SW, TS, DS, and JS.

### Conflict of Interest Statement

The authors declare that the research was conducted in the absence of any commercial or financial relationships that could be construed as a potential conflict of interest.

## Footnotes

^1^https://www.nps.gov/articles/understanding-fire-danger.htm

^2^https://www.porkbusiness.com/article/usda-estimates-number-us-swine-operations-farms
