# Supplementary material for: Willingness to Comply With Biosecurity in Livestock Facilities: Evidence From Experimental Simulations
Source: Front Vet Sci. 2019 Jun 4;6:156. doi: 10.3389/fvets.2019.00156 (PMC6558082; doi:10.3389/fvets.2019.00156)
Supplement: Supplementary file 1 [file Data_Sheet_1.PDF]

Supplemental Material Appendix A: Model Selection

We used an information-theoretic approach for candidate model selection for both experiments (Burnham and Anderson, 2002; Burnham and Anderson, 2004). We developed a set of candidate models based on the treatments and variables presented above with the probability of complying with the shower facility as the response variable. Candidate models were assessed to measure the relative information explained by each model using Akaike's Information Criterion (AIC) (Akaike, 1973; Burnham and Anderson, 2002; Burnham and Anderson, 2004) and each model's ΔAIC, where the ΔAIC can be used to calculate the probability that the selected candidate model is the best model within the selected candidate model set, given the data. For example, Model 1 used the independent variables Diagnosis Certainty, Message Delivery Method, Infection Risk, Psychological Distance and the interaction terms Message Delivery Method by Infection Risk and Message Delivery Method by Diagnosis Certainty, as well as a random participant effect. Model 1's ability to explain variation in the binary dependent variable, use of the Shower in – Shower out facility, was quantified using AIC. This process was repeated for each of the candidate models listed in Tables S1 (Experiment One) and S2 (Experiment Two) and one model with the lowest AIC value was selected for inference from each experiment. See Tables S1 and S2 for candidate model composition.

Experiment One Model Selection

The AIC-selected best candidate model was Model 8 (Table 3). Model 7 had only a slightly higher AIC value but was discarded because it was identical to Model 8 with the exclusion of the Play Order variable. Other models did not have significant support for being the best candidate model (Table 3). All inference reported for Experiment One was generated using Model 8.

Table S1. Candidate Models for Experiment One reordered by AIC value with the best AIC-selected models listed first.

	Fixed Effect Variables										
**Model**	**DC**	**PD**	**M**	**IR**	**PO**	**M*IR**	**IR*DC**	**M*DC**	**M*DC*IR**	**AIC**	**ΔAIC**
Model 8	x	x	x	x	x	x	x	x	x	2419.63	0
Model 7	x	x	x	x		x	x	x	x	2420.19	0.563
Model 5	x	x	x	x	x	x	x			2425.03	5.404
Model 2	x	x	x	x		x	x			2425.85	6.219
Model 6	x	x	x	x		x	x	x		2426.32	6.696
Model 3	x	x	x	x			x	x		2440.88	21.256
Model 1	x	x	x	x		x				2532.57	112.947
Model 4	x	x	x	x		x		x		2534.31	114.682

Participant was a random effect in all models. Fixed effects included DC = Diagnosis Certainty, PD = Psychological Distance, IR = Infection Risk, M = Message Delivery Method, and PO = Play Order. AIC is Akaike's Information Criterion. The model with the lowest AIC is considered the model with the most support for being the best candidate model. ΔAIC is a measure of how much information is lost if a different candidate model is selected for inference.

Experiment Two Model Selection

The AIC-selected best candidate model for Experiment Two was candidate Model 2 (Table S2). Model 5 was discarded because it simply added the variable Play Order and did not improve the overall fit to the data using a chi-squared test for goodness of fit ($\chi^2(1) = 1.397$ p= 0.2372). Similarly, Models 6 and 7 had support for being good models, yet differentiated themselves from Model 2 through the addition of one or two interaction terms (Model 6: Message by Contagion Certainty. Model 7: Message by Contagion Certainty and the three-way interaction term: Message* Contagion Certainty *Infection Risk).

Table S2. Candidate Models for Experiment Two reordered by AIC value with the best AIC-selected models listed first.

	Fixed Effect Variables											
**Model**	**CC**	**PD**	**M**	**IR**	**A**	**PO**	**M*IR**	**IR*CC**	**M*CC**	**M*CC*IR**	**AIC**	**ΔAIC**
Model 2	x	x	x	x	x		x	x			2843.5	0
Model 5	x	x	x	x	x	x	x	x			2844.7	1.247
Model 6	x	x	x	x	x		x	x	x		2847.1	3.59
Model 7	x	x	x	x	x		x	x	x	x	2851.3	7.829
Model 8	x	x	x	x	x	x	x	x	x	x	2852.5	9.006
Model 3	x	x	x	x	x			x			2871.7	28.157
Model 1	x	x	x	x	x		x				2887.3	43.762
Model 4	x	x	x	x	x		x		x		2890.6	47.103

Participant was a random effect in all models. Fixed effects included CC = Contagion Certainty, PD = Psychological Distance, IR = Infection Risk, M = Message, PO = Play Order, A= Audience (AMT or In-house). AIC is Akaike’s Information Criterion. The model with the lowest AIC is considered to be the model with the most support for being the best candidate model. ΔAIC is a measure of how much information is lost if a different candidate model is selected for inference.
